# Health-related quality of life assessment in eating disorders: adjustment and validation of a specific scale with the inclusion of an interpersonal domain

**DOI:** 10.1007/s40519-020-01081-5

**Published:** 2020-12-14

**Authors:** Paolo Meneguzzo, Patrizia Todisco, Sofia Calonaci, Cecilia Mancini, David Dal Brun, Enrico Collantoni, Lorenzo Maria Donini, Elena Tenconi, Angela Favaro

**Affiliations:** 1 Department of Neuroscience, University of Padova, via Giustiniani 2, 35128 Padua, Italy; 2 Eating Disorders Unit, Casa Di Cura “Villa Margherita”, Arcugnano, VI Italy; 3 Department of Linguistic and Literary Studies, University of Padova, Padua, Italy; 4 Experimental Medicine Department, Food Science and Human Nutrition Research Unit, Sapienza University of Rome, Rome, Italy; 5 Padova Neuroscience Center, University of Padova, Padua, Italy

**Keywords:** Eating Disorders, Health-related quality of life, Anorexia Nervosa, Bulimia Nervosa, Binge Eating Disorder, Interpersonal domain

## Abstract

**Purpose:**

Quality of life is a fundamental aspect of both clinical practice and research on eating disorders (ED) due to the significant impacts these disorders have on everyday life. Disorder-specific scales can improve the quality of research and findings and offer greater sensitivity and responsiveness. However, no specific instrument is available in Italian for ED. The aim of this paper is to adjust and to validate a reliable scale with specific items regarding physical and interpersonal well-being.

**Methods:**

The Italian version of the Eating Disorder Quality of Life (IEDQOL) scale was developed, on the basis of the original English scale, with the addition of items pertaining to physical well-being and interpersonal interactions. In this study, 180 ED patients and 190 healthy controls from the community were enrolled both from inpatient units and outpatient services. A statistical analysis with an exploratory factorial approach was performed in order to validate the tool.

**Results:**

The results showed that the IEDQOL has very good psychometric properties with test–retest validity and sensitivity between patients and controls (*d* = 2.17 for total score). Moreover, the interpersonal domain showed excellent psychometric values (Cronbach’s *α* > 0.70 in all the subgroups) and a robust correlation with other quality of life constructs.

**Conclusion:**

Future studies on the Italian population should use IEDQOL as outcome element that can be useful also with other disorder-specific psychopathological constructs and corroborate the reliability of the data. Future research in the ED field should only use this specific tool.

**Level of evidence:**

Case–control analytic study, Level III.

## Introduction

Eating disorders (ED) are one of the most common health problems among adolescents and young adults in Western countries, with the highest mortality rate among all mental illnesses [1–5]. They are characterized by negative beliefs regarding body shape and weight and by irregular eating habits, such as restricted eating, binge eating and compensatory behaviors (e.g., vomiting, fasting and laxative abuse). They include anorexia nervosa (AN), bulimia nervosa (BN), binge eating disorder (BED) and other specified feeding and eating disorders (OSFED). AN is characterized by an inadequate food intake and the inability to maintain a healthy weight. BN is characterized by recurrent episodes of binge eating, followed by unhealthy compensatory behaviors. BED is marked by frequent episodes of binge eating without purging behaviors. OSFED include patients with symptoms that do not fully meet the diagnostic criteria of AN, BN or BED [6]. ED have a common psychopathology, characterized by depressive and anxiety symptoms, interpersonal sensitivity and a sense of ineffectiveness [7]. All of them are more common in females, even though they are characterized by different onset ages as well as different clinical trajectories [8]. Psychiatric comorbidities and medical complications are common in ED, and may involve various organs and systems; in addition, they may be linked to undernutrition, purging or overweight [8–12]. Furthermore, patients preset psychological difficulties that can impair social aspects of everyday life (e.g., school, work and relationships), and could have a relevant impact on life experiences [13].

The symptoms of ED have significant impacts on people that are suffering from these conditions, and a recent meta-analysis has shown that their quality of life (QoL) measures are significantly lower than those of the healthy population, with significant economic burdens [14, 15]. Quality of life can be defined as a broad concept that encompasses an individual’s satisfaction with his/her health state and functioning in the physical, psychological, social, and cognitive domains [16–18]. It has been reported in the literature that QoL is correlated with ED severity [19] and also has a significant impact in overweight patients [9, 20–22]. Findings also suggest that an assessment of the QoL is needed in treatment research, with a specific focus on the construct called health-related quality of life (HRQoL), which overlaps with QoL in the physical, social and mental health dimensions [23, 24]. The literature showed that QoL in ED has been assessed primarily with four ED-specific instruments, as well as with generic scales, but without a specific rationale for the choice [25]. To our knowledge, there are four ED-specific QoL scales: the eating disorders quality of life instrument (EDQOL), a 25-item self-report questionnaire that covers four domains [26]; the eating disorders quality of life scale (EDQLS), a 40-item self-report questionnaire that covers 12 domains [27]; the health-related quality of life in eating disorders questionnaire (HeRQoLEDv2), a 50-item self-report questionnaire that covers eight domains [28]; and the quality of life for eating disorders (QOLED), a 20-item self-report questionnaire with six domains [29]. Researches found few if any differences in the QoL among the four diagnostic groups using generic questionnaires for the assessment, such as the Short Form-36 health survey [19, 30, 31]. Other studies, such as the review from Jenkins et al. [23], observed some differences in QoL among the diagnostic groups, with a tendency towards lower scores in the BED sample, and the authors underline that the use of a generic QoL scale can be a bias for the AN subsample. Moreover, a recent meta-analysis [32] suggests that no significant differences among EDs could be defined by generic HRQoL scales, and advocated for the implementation of disorder-specific HRQoL scale in clinical research. Indeed, clinical studies showed that, although HRQoL has been shown to improve after treatment, these ED patients were still more dysfunctional than those with other psychiatric illnesses (such as severe depression) and the rest of the population [19, 33–35]. However, these results were obtained with generic QoL or HRQoL scales, which may be unable to detect significant changes of specific aspects of the patients’ lives, as demonstrated by previous meta-analyses [23, 32].

Interventions that specifically tackle QoL in the treatment of ED patients would be useful because improving QoL may contribute to the resolution of ED symptoms; besides, simply eliminating ED symptoms may not completely resolve the poor QoL reported by patients [36, 37]. Further investigations are needed to better detect the potential differences in QoL among the diagnostic categories, especially with culturally and linguistically appropriate tools [38, 39]. Plus, there has been a recent expansion in the literature analyzing the role of interpersonal difficulties in ED and the impact this impairment may have upon the patients’ everyday lives and disorders [40–42]. The English version of the HRQoL for ED [26] does not include a set of items pertaining to the interpersonal domain, and other ED-HRQoL scales appear to be more time-consuming [27, 28], or need to be evaluated with a more rigorous methodology [43, 44].

Given the importance of the HRQoL measurements in the treatment and recovery of ED patients, it is critical for clinicians to be able to assess these dimensions with specific and robust tools. To date, there is no Italian-language tool to comprehensively assess the QoL of individuals with a diagnosis of an ED, and previous Italian studies used a general scale for the evaluation [33, 37]. Moreover, there is a growing body of evidence that the interpersonal domain in ED plays a key role in the specific psychopathology, and therefore should be included in the QoL assessment [7, 45]. Thus, the aim of this study is to develop and validate a QoL questionnaire, which is suitable for Italian patients with an ED, as well as suggest a revision of international HRQoL scales for ED with the implementation of an interpersonal subscale and advocate for future research with disorder-specific tools.

## Materials and methods

### Study design

This is a multi-center cross-sectional study, which consists of a collection of data from a group of healthy controls (HCs) and a group of patients with at least one of the following ED: AN, BN, BED, and OSFED.

### Sample and setting

The study was conducted between January 2019 and January 2020 at the Eating Disorder Center of the University Hospital of Padova (Italy) and at the Eating Disorders Unit of the Casa di Cura “Villa Margherita” in Arcugnano (Vicenza, Italy). All patients were admitted to the outpatient and inpatient clinics and were assessed for the inclusion criteria: (a) any eating disorder diagnosis; (b) 14 years of age or older; (c) the ability to understand and fill out the questionnaires. The HC were enrolled using public announcements and ads on social media and in the authors’ social networks. The HC were screened for any ED and psychiatric disorder history by a trained researcher using the structured clinical interview for DSM-5 [46]. An informed consent to participate was signed by all subjects or by legal representatives, if subjects were underage, and all the information was collected anonymously. The ethical committee’s approval was not required for this kind of survey as per local legislation and national guidelines.

### Instruments

The Italian translation of the Eating Disorders Quality of Life (IEDQOL) measure was carried out on the basis of the Eating Disorders Quality of Life (EDQOL) measure, a HRQoL tool published by Engel et al. [26]. The original scale comprises 25-items and 4 subscales: psychological, physical/cognitive, financial, school/work. The tool showed excellent psychometric proprieties and a higher sensitivity than generic tools [26]. The choice of this starting scale was made upon the basis of the consensus among the authors who conceptualized the study (PM, PT, LMD, ET, AF), and it was based on the number of items and the clarity and formal simplicity of the scale. The translation of the EDQOL into Italian was carried out according to the recent methodological recommendations [47]:

1. The translation from the original English version into Italian was carried out by two independent authors (PM and DDB) who are both Italian native speakers with English as their first foreign language.
2. The two Italian versions were reviewed by a group of Italian native speakers who are experts with multidisciplinary backgrounds (PT, CM, ET, AF), and a final consensus version was obtained.
3. A backward translation was performed by a professional bilingual translator (DDB) in order to evaluate the semantic value of the questionnaire.
4. A feasibility evaluation was performed with a small sample of patients (10 subjects) in order to evaluate the applicability of the questionnaire within the framework of a structured interview, and comments and observations were made.
5. A quantitative evaluation of the IEDQOL was performed in clinical and control groups, using an exploratory factorial analysis for the validation of the scale and correlation analyses for the comparison with previously validated instruments.

However, in the authors' opinion, the translated scale showed gaps in the assessment of clinical and social impacts of ED. Indeed, the interpersonal domain is totally absent in the original scale and the physical comorbidities investigation is limited to few items, but ED have a significant impact on the patients’ body [48]. Three different authors (PM, PT, ET) separately created a pool of 10 items per-person regarding physical and interpersonal domains, which were rated by the other authors for linguistic valence and adequacy for patients with ED. The final consensus of all authors was obtain for 10 items that got the best assessment. The other items were dismissed because of redundancies or because they were off topic. Therefore, ten items were added to the original 25-item EDQOL, in order to improve the evaluation of the physical impact of the disorders and the interpersonal domain, which are important aspects of the patients' lives [48, 49].

For the validation of the scale, IEDQOL was compared to Italian standardized questionnaires, namely the inventory of interpersonal problems (IIP-32), which is broadly used to measure both interpersonal and social functioning/abilities in the general population [50]; the eating disorder examination questionnaire (EDE-Q), which is broadly utilized to assess eating disorder psychopathology [51]; the sense of coherence (SOC), a questionnaire used to measure the ability to successfully employ one’s resources in the management of life stressors [52]; the health survey short form (SF-12), which was compared to the general quality of life measurement [53]; the brief symptom inventory (BSI-53), a well-known scale used to evaluate psychological distress and psychiatric symptomatology [54]; and, finally, the patient health questionnaire (PHQ9), which was utilized as a specific depression measurement tool, since depression is a significant comorbidity in the ED population [55].

### Statistical analyses

The socio-demographic and clinical variables were described by descriptive statistics such as mean, standard deviation and range where indicated. In a scale adaptation, the validity of the structure must be tested because both the translation process, and the addition of new items, could modify the latent variables [56]. Therefore, the psychometric proprieties of the IEDQOL were assessed by applying an exploratory factor analysis (EFA) using Varimax rotation with Kaiser normalization. Internal consistency was evaluated by Cronbach's *α* index. Test–retest analysis was performed using the intraclass correlation coefficient (ICC) in a subsample that completed a randomized-item retest one week after. Correlation analyses were performed employing Pearson’s approach. Analyses between groups were run via an ANOVA analysis. Linear regression analysis was used to evaluate the relationship between the QoL of the patients and the severity of the ED. All analyses were performed with the IBM SPSS Statistics 25.0 software (SPSS, Chicago, IL, USA).

## Results

A total of 370 subjects participated in the evaluation: 180 ED patients and 190 HCs. Seventy-one patients had a diagnosis of AN (39.4%), 46 patients had BN (26.1%), 33 had BED (18.3%) and 30 had OSFED (16.6%). Seventy-six patients (42.2% of the total sample) were recruited at the Eating Disorder Unit of Villa Margherita at the beginning of their inpatient treatment. The socio-demographic characteristics of the sample are reported in Table 1. Clinical scores for depression were found in the clinical sample: PHQ9 ED = 13.3 (± 6.41); HC = 5.35 (± 4.31); no difference in depression scores was found between the inpatient and outpatient samples. The differences in the scores between the ED groups and HCs were as expected: the ED groups experienced more eating psychopathology (EDE-Q global score ED = 3.39 ± 1.47, HC = 0.95 ± 1.05), lower QoL (mental subscale of the SF12 ED = 33.49 ± 10.36, HC = 44.46 ± 11.92), less sense of coherence (SOC total score ED = 50.00 ± 12.98, HC = 65.64 ± 14.98) and higher interpersonal problems (e.g., IIP32 controlling scale ED = 3.55 ± 2.98, HC = 1.74 ± 2.51; IIP32 distant subscale ED = 7.14 ± 4.15, HC = 2.69 ± 2.49). Furthermore, there were no missing or multiple answers, and no problems in comprehension emerged.

**Table 1:** Demographic characteristics

	Total (*n* = 370)	AN (*n* = 71)	BN (*n* = 46)	BED (*n* = 33)	OSFED (*n* = 30)	HC (*n* = 190)
Age, years (SD)	26.16 (9.29)	25.16 (9.25)	26.54 (10.74)	32.62 (13.22)	27.21 (11.48)	25.22 (7.20)
Range	[14–60]	[14–50]	[16–60]	[15–55]	[18–54]	[16–60]
BMI, kg/m^2^ (SD)	22.51 (6.64)	15.75 (1.52)	22.33 (2.86)	36.18 (9.02)	26.58 (7.29)	22.06 (2.96)
Range	[12.07–57.87]	[12.07–18.50]	[18.02–30.80]	[24.03–57.87]	[18.14–38.87]	[18.04–35.66]
Female (%)	96.22	100.00	95.65	93.94	100.00	94.74
Civil status						
Single (%)	85.41	90.10	52.70	68.75	90.00	86.32
Married (%)	13.24	8.45	10.87	28.21	10.00	13.68
Educational level						
Middle school (%)	15.68	33.80	28.26	27.27	26.66	2.10
High school (%)	40.27	50.70	34.78	60.60	46.66	33.15
University (%)	44.05	15.50	36.96	12.13	26.68	64.75
Living with others (%)	91.62	94.37	84.78	87.87	100.00	91.58
Duration of ED, years (SD)	5.35 (5.02)	5.92 (5.43)	5.16 (6.11)	5.67 (3.91)	3.96 (2.43)	–

*SD* standard deviation, *AN* anorexia nervosa, *BN* bulimia nervosa, *BED* binge eating disorder, *OSFED* other specified feeding or eating disorder, *HC* healthy control

### Feasibility evaluation

From the feasibility evaluation, the structure of items and their comprehension was confirmed. Indeed, none of the patients reported difficulty in understanding the items, and all of them found the questionnaire tolerable. Interpersonal items were found adequate in reverse form by 8 patients out of 10. Comments about the structure of the phrases were taken into consideration and the final items were approved by all the authors.

### Exploratory factor analysis

According to Engel et al. [26], each item was scored from 0 (never) to 4 (always) on a Likert scale. Only 3 new items regarding interpersonal relationships were chosen as reverse items with an opposite score scale. The EFA analysis was performed on all the samples in accordance with the original validation of the EDQOL [26] and with no prior rationale about the new items added. Indeed, the purpose of our study was to validate an Italian ED-specific QoL scale; thus, the entire spectrum of the disorders, from outpatients to inpatients and controls, had to be included. The EFA yielded a Kaiser–Meyer–Olkin value of 0.949 and a Bartlett's Chi-square of 9912.715 (*p* < 0.001). Five factors were generated, which explains the 70.68% variance. Table 2 summarizes the factor analysis. Three items showed meaningful cross loading between two factors, but the correlation coefficient between each item with the total of its hypothesized domain was over 0.50 (Pearson’s correlation coefficient) in all the items and for all the domains. On the other hand, two items showed low consistency with all the factors and were subsequently deleted. The deleted items referred to sleep satisfaction and own-house pride. For the remaining items, the discriminant validity was supported by the correlation of each item with its hypothesized factor, which was higher than its correlation with other domains. The item–total correlation analyses showed that all the included items resulted in a score higher than 0.30, as is desirable [57].

**Table 2:** Exploratory factor analysis

IEDQOL	Factor 1	Factor 2	Factor 3	Factor 4	Factor 5	*h*^2^	Item–total correlation
Feel embarrassed	0.836					0.795	0.781
Feel worse about self	0.839					0.810	0.800
Want to avoid people	0.767					0.772	0.822
Not get better	0.789					0.783	0.804
Feel lonely	0.809					0.836	0.848
Less interest/pleasure	0.770					0.800	0.852
Not care about self	0.697					0.690	0.781
Feel odd	0.789					0.765	0.802
Avoid eating in front of others	0.715					0.683	0.767
Discussions of problems with relatives	0.696					0.711	0.787
Cold feet/hands		0.631		0.401		0.632	0.671
Headache		0.531				0.670	0.718
Weakness		0.621				0.741	0.750
Loss hair		0.636				0.578	0.556
Tooth or gum problems		0.649				0.547	0.358
Bad digestion		0.574				0.530	0.567
Worried about weight	0.496	0.804				0.722	0.723
Attention		0.454				0.776	0.821
Understand of information		0.463				0.692	0.821
Focusing		0.539				0.787	0.710
Cost problems			0.712			0.642	0.819
Difficulty paying bills			0.837			0.738	0.379
Significant financial debt			0.859			0.778	0.395
Need to spend/credit card			0.664			0.646	0.582
Need to borrow money			0.725			0.597	0.414
Leave of absence	0.454			0.737		0.811	0.751
Low grades				0.648		0.673	0.676
Reduce work hours				0.695		0.819	0.766
Lose job				0.748		0.688	0.569
Failure in class				0.575		0.480	0.349
Friends					0.522	0.502	0.476
Sentimental relationship					0.864	0.866	0.488
Sexual relationship					0.858	0.863	0.510
Own house						0.413	0.340
Sleep						0.351	0.289
% of variance accounted for after rotation	27.728	14.023	11.214	10.685	7.026		

*IEDQOL* italian eating disorders quality of life. Factor loads > 0.40 are shown. *h*^2^ communality

### Subscale properties

According to the EFA, the scale consists of 5 factors, and they were consistent with the original 4 subscales with the addition of a new one. The factors were labeled as in the original scale. Factor 1 was labeled ‘psychological subscale’ because the items are related to feelings and emotions. Factor 2 was labeled as ‘physical/cognitive subscale’ because the items refer to physical symptoms and cognitive functioning. Factor 3 was labeled ‘financial subscale’ because its items all pertain to the economic impact of the ED. Factor 4 was labeled ‘school/work subscale’ because its items describe difficulties with school outcomes or with work. Factor 5 was labeled ‘interpersonal subscale’ because its items are related to the interpersonal domain. The skewness coefficients for subscales were 0.182 for IEDQOL psychological, 0.633 for IEDQOL physical-cognitive, 3.049 for IEDQOL financial, 1.600 for IEDQOL work/school, 0.063 for IEDQOL interpersonal and 0.521 for IEDQOL total score. Thus, except for the financial subscale, the IEDQOL subscales showed an approximate symmetry or a moderate skew.

### Internal consistency and test–retest reliability

Cronbach’s α coefficients for all the domains were greater than 0.70 in all the subsamples (see Table 3). The test–retest stability was performed with the ICC test using a subsample of 30 patients and 20 controls who agreed to perform a 1-week retest. All the ICC values exceeded 0.90, which indicates there was significant stability across time.

**Table 3:** Internal consistencies and intercorrelations in the subsamples and subscales

	*α*	IEDQOL-P	IEDQOL-PC	IEDQOL-F	IEDQOL-WS	IEDQOL-I
Anorexia nervosa						
IEDQOL-P	0.884	–				
IEDQOL-PC	0.821	0.657**	–			
IEDQOL-F	0.829	0.308**	0.280**	–		
IEDQOL-WS	0.820	0.555**	0.588**	0.390**	–	
IEDQOL-I	0.755	0.208*	0.113*	0.369**	0.335**	–
IEDQOL-TS	0.920	0.860**	0.843**	0.541**	0.798**	0.392**
Bulimia nervosa						
IEDQOL-P	0.935	–				
IEDQOL-PC	0.852	0.746**	–			
IEDQOL-F	0.821	0.298*	0.319*	–		
IEDQOL-WS	0.805	0.701**	0.511**	0.336**	–	
IEDQOL-I	0.704	0.287*	0.110*	0.114*	0.189*	–
IEDQOL-TS	0.934	0.935**	0.867**	0.470**	0.760**	0.330*
Binge eating disorder						
IEDQOL-P	0.825	–				
IEDQOL-PC	0.924	0.713**	–			
IEDQOL-F	0.751	0.465*	0.280	–		
IEDQOL-WS	0.710	0.289*	0.473**	0.596**	–	
IEDQOL-I	0.761	0.225*	0.819*	0.635**	0.280*	–
IEDQOL-TS	0.932	0.909**	0.881**	0.503**	0.599**	0.567**
OSFED						
IEDQOL-P	0.816	–				
IEDQOL-PC	0.814	0.790**	–			
IEDQOL-F	0.894	0.529**	0.544**	–		
IEDQOL-WS	0.864	0.421*	0.364*	0.719*	–	
IEDQOL-I	0.869	0.396*	0.368*	0.312*	0.360*	–
IEDQOL-TS	0.909	0.939**	0.911**	0.644**	0.481*	0.551*
Healthy control						
IEDQOL-P	0.936	–				
IEDQOL-PC	0.832	0.666**	–			
IEDQOL-F	0.805	0.360**	0.484**	–		
IEDQOL-WS	0.798	0.510**	0.602**	0.674**	–	
IEDQOL-I	0.756	0.351**	0.330**	0.155**	0.310**	–
IEDQOL-TS	0.927	0.902**	0.875**	0.533**	0.694**	0.542**

*α* Cronbach’s *α*, *IEDQOL* Italian eating disorders quality of life, *P* psychological, *PC* physical/cognitive, *F* financial, *WS* work/school, *I* interpersonal, *TS* total score

*p < 0.05

**p < 0.001

### Concurrent and convergent validity

Tables 3 and 4 display the Pearson bivariate correlation coefficients between IEDQOL and the other scales used in the study for well-being, psychopathology and QoL. Concurrent validity was determined by correlation analysis, with the correlation coefficients showing significant correlations with all the measures. In particular, the data showed a high correlation between the EDE-Q total score and IEDQOL total score (*r* = 0.858, *p* < 0.001), as well as between the IDEQOL subscales and the SF-12 subscales (convergent validity).

**Table 4:** Pearson correlation between IEDQOL and construction-related instruments in the total sample

	IEDQOL-P	IEDQOL-PC	IEDQOL-F	IEDQOL-WS	IEDQOL-I	IEDQOL-TS
IIP32						
PA	0.300**	0.280**	0.315**	0.319**	0.257**	0.349**
BC	0.485**	0.457**	0.402**	0.393**	0.312**	0.512**
DE	0.578**	0.577**	0.490**	0.515**	0.518**	0.653**
FG	0.500**	0.494**	0.282**	0.411**	0.419**	0.542**
HI	0.504**	0.510**	0.304**	0.440**	0.336**	0.548**
JK	0.408**	0.447**	0.232**	0.380**	0.333**	0.463**
LM	0.508**	0.528**	0.291**	0.425**	0.360**	0.544**
NO	0.240**	0.197**	0.175**	0.195**	0.137**	0.225**
EDE-Q						
Restraint	0.732**	0.698**	0.393**	0.584**	0.352**	0.740**
Eating concern	0.807**	0.731**	0.457**	0.627**	0.453**	0.813**
Shape concern	0.852**	0.722**	0.451**	0.613**	0.465**	0.831**
Weight concern	0.838**	0.718**	0.447**	0.629**	0.410**	0.818**
Global score	0.866**	0.767**	0.468**	0.657**	0.450**	0.858**
SOC						
Comprehensibility	− 0.595**	− 0.548**	− 0.379**	− 0.461**	− 0.540**	− 0.642**
Manageability	− 0.625**	− 0.562**	− 0.364**	− 0.477**	− 0.472**	− 0.643**
Meaningfulness	− 0.639**	− 0.580**	− 0.479**	− 0.506**	− 0.515**	− 0.678**
Total score	− 0.683**	− 0.622**	− 0.451**	− 0.531**	− 0.565**	− 0.721**
SF-12						
PCS-12	− 0.510**	− 0.556**	− 0.422**	− 0.374**	− 0.229**	− 0.566**
MCS-12	− 0.619**	− 0.596**	− 0.322**	-0.550**	− 0.569**	− 0.671**
PHQ total score	0.760**	0.758**	0.490**	0.604**	0.494**	0.808**
BSI-GSI	0.745**	0.754**	0.435**	0.649**	0.503**	0.808**
Age	0.008	0.21	0.165**	− 0.159**	− 0.104*	− 0.031
BMI	0.081	− 0.150**	0.008	− 0.191**	− 0.040	− 0.056

*IEDQOL* Italian Eating Disorders Quality of Life, *P* psychological, *PC* physical/cognitive, *F* financial, *WS* work/school, *I* interpersonal, *TS* total Score, *IIP32* Inventory of Interpersonal Problems, *IIP33-PA* Domineering/Controlling subscale, *IIP32-BC* Vindictive/Self-centered subscale, *IIP32-DE* cold/ distant subscale, *IIP32-FG* socially inhibited/avoidant subscale, *IIP32-HI* non-assertive subscale, *IIP32-JK* overly accommodating/exploitable subscale, *IIP32-LM* self-sacrificing/overly nurturant subscale, *IIP32-NO* intrusive/needy subscale, *EDE-Q* eating disorder examination questionnaire, *SOC* sense of coherence, *SF-12* short form of health survey, *SF12-PCS* physical score short form, *SF12-MCS* mental health short form, *PHQ* patient health questionnaire, *BSI-GSI* Brief Symptom Inventory—Global Severity Index, *SD* standard deviation

**p < 0.001

### Sensitivity analysis in different populations

Compared to the general population, patients with ED were significantly more impaired in all domains. Table 5 shows means and standard deviations for all groups included in the study. The clinical samples scored higher than HCs in all subscales of the IEDQOL and AN patients showed the highest scores in all subscales among the clinical groups. The effect size analyses ranged from 0.79 for the financial domain to 2.29 for the psychological domain; that is, there were large effect sizes in all the domains. Moreover, the analysis of the ED subgroups yielded significant differences.

**Table 5:** IEDQOL scores in the included sample. Means and standard deviations are represented

	ED	HC	*t*	*p*	*d*	AN	BN	BED	OSFED	*F*	*p*	Post hoc
IEDQOL-P	2.48 (0.84)	0.67 (0.74)	21.928	0.000	2.29	2.55 (0.80)	2.36 (0.98)	2.27 (0.81)	2.68 (0.70)	122.619	0.000	
IEDQOL-PC	1.84 (0.86)	0.59 (0.56)	16.474	0.000	1.72	2.17 (0.74)	1.77 (0.83)	1.40 (0.97)	1.65 (0.79)	87.765	0.000	AN > BN AN > BED AN > OSFED
IEDQOL-F	0.48 (0.74)	0.05 (0.21)	7.585	0.000	0.79	0.56 (0.72)	0.45 (0.66)	0.23 (0.47)	0.60 (1.04)	18.063	0.000	AN > BED
IEDQOL-WS	1.09 (1.01)	0.09 (0.29)	12.565	0.000	1.01	1.54 (1.05)	0.90 (0.09)	0.52 (0.68)	0.91 (1.02)	63.435	0.000	AN > BN AN > BED AN > OSFED
IEDQOL-I	2.31 (1.03)	1.29 (0.98)	9.811	0.000	1.02	2.69 (0.85)	2.05 (0.95)	1.93 (1.13)	2.22 (1.16)	29.818	0.000	AN > BN AN > BED
IEDQOL-TS	1.76 (0.67)	0.52 (0.45)	20.449	0.000	2.17	2.00 (0.62)	1.65 (0.67)	1.35 (0.62)	1.73 (0.62)	125.986	0.000	AN > BN AN > BED

*IEDQOL* Italian Eating Disorders Quality of Life, *P* psychological, *PC* physical/cognitive, *F* financial, *WS* work/school, *I* interpersonal, *TS* total score, *ED* eating disorder patients, *HC* healthy controls, *AN* anorexia nervosa, *BN* bulimia nervosa, *BED* binge eating disorder, *OSFED* other specified feeding and eating disorders, *t* t test, *F* ANOVA, *d* Cohen’s *d*, Post hoc Bonferroni correction

## Discussion

The goal of this study was to adapt an easy and short tool for the assessment of the QoL of patients with ED in the Italian population, and to prompt the value of the inclusion of an interpersonal domain in the HRQoL scales for ED. According to the literature, it is preferable to select a validated tool and alter it for cultural adaptation rather than create a new one [58]. Clinicians and researchers should apply a HRQoL instrument specifically tailored to the ED psychopathology, especially when it comes to substantiating outcome measurements or changes due to specific interventions; such a tool could additionally be used to facilitate communication among different centers or healthcare workers, and increase outcome data reliability.

The analysis showed valid and stable results from our samples, confirming the original four-factor structure of the EDQOL [26] and adding an interpersonal subscale. Internal consistency and test–retest validity produced good to excellent results for quality of life assessment, as suggested by the literature [59]. The new items included did not influence the structure of the questionnaire but, instead, added a fundamental construct in ED, i.e., interpersonal relationships [60, 61]. Indeed, patients reported social support as one of the most important aspects of QoL, and social interactions are one of the triggering factors of pathological behaviors, increasing the relapse rates and compromising recovery outcome [62, 63].

Differences among the subgroups were in line with previous studies which showed a lower health-related QoL in patients, compared with controls [32]. As for the ED subgroups, the literature presents mixed results, but our data corroborated the findings that reported a lower QoL in AN patients compared with other clinical groups [19, 29, 31]. This is consistent with the physical and cognitive impairment showed by AN patients and with the severe impairment of their life trajectories [14]. Moreover, our data confirmed the direct relationship between high levels of eating psychopathology and low QoL and a relationship with BMI only for specific domains (physical/cognitive and work/school), as already demonstrated [64]. However, according to the literature, QoL should not be the main focus of treatment; it should be taken into account in order to achieve a new balance between cognitive-behavioral symptoms and everyday life [65]. Age only had a small correlation with the school/work subscale, showing that IEDQOL could be used in a 14 year olds to 60 year olds population. Moreover, IEDQOL subscales showed good correlations with SF-12 subscales, which proves their usefulness in the evaluation of HRQoL with a high potential in the discrimination between clinical and non-clinical populations.

The interpersonal domain showed a significant positive correlation with eating psychopathology, corroborating the relationships between the impairment in interpersonal social skills and the severity of the disorder [66]. Indeed, interpersonal impairment could lead to a higher expression of emotions with an adverse impact on the outcome of treatments [67]. The presence of a specific interpersonal subscale in the clinical evaluation of patients could be used to address the treatment into interpersonal domains and tailor a treatment for specific ED profiles [68]. Thus, the idea of including an investigation of the interpersonal domain has been positively supported by our data, and the IEDQOL scale could be considered as an important example for translations into other languages.

The current study provides the groundwork for future research, but it still presents several limitations. Firstly, the typical limitations and advantages of using self-administered questionnaires should be considered [69]. Secondly, the test–retest analysis was performed on a small sample of patients and controls, and future research could possibly investigate larger samples. Despite these limitations, this tool has the potential to be an effective transdiagnostic device for clinical applications due to the presence of all ED subtypes in this validation study.

## Conclusion

The interpersonal domain has showed a very good reliability and correlation with the other subscales, confirming the relevance of this construct for the evaluation of ED-related psychological impairment. The IEDQOL scale appears to be a promising disorder-specific tool, and this study shows that it is a reliable and valid tool for the measurement of QoL in Italian ED patients. The scale may have various applications, both in clinical and research settings, as well as improve the description of the outcomes of patients who still have low rates of recovery. The addition of the interpersonal domain assessment is a valid improvement for ED HRQoL scales. Further research, including independent validation studies, is recommended. Besides, our results showed that a specific ED tool is able to highlight QoL differences among clinical subgroups.

The IEDQOL scale is available in the Supplementary Material of the paper and from the corresponding author.

### What is already known on this subject?

Health-related quality of life (HRQOL) is a multidimensional construct that reflects the degree to which an individual is healthy, comfortable, and able to enjoy life events, and should be studied with specialized psychometric tools in a specific disorder. It is significantly compromised in eating disorder patients, due to the severe impact of the disorder on everyday life, and it is linked to the subjects’ cognitive and behavioral routines, as well as interpersonal relationships.

### What this study adds?

This study underlines the importance of the interpersonal domain in the patients’ quality of life, and advocates for the implementation of the international HRQOL scale for ED with a specific domain. Moreover, this paper aims to validate a specific and significant tool for the assessment and the outcome evaluation for the Italian ED population.
